# A Central Role for STAT3 in Gammaherpesvirus-Life Cycle and -Diseases

**DOI:** 10.3389/fmicb.2016.01052

**Published:** 2016-07-08

**Authors:** Xiaofan Li, Sumita Bhaduri-McIntosh

**Affiliations:** ^1^Pediatric Infectious Diseases, Department of Pediatrics, Stony Brook University School of MedicineStony Brook, NY, USA; ^2^Department of Molecular Genetics and Microbiology, Stony Brook University School of MedicineStony Brook, NY, USA

**Keywords:** gammaherpesvirus, Epstein-Barr virus, Kaposi's sarcoma-associated herpesvirus, STAT3, latency, lytic cycle, viral persistence, DNA damage response

## Abstract

Having co-evolved with humans, herpesviruses have adapted to exploit the host molecular machinery to ensure viral persistence. The cellular protein Signal Transducer and Activator of Transcription 3 (STAT3) is a leading example. STAT3 is a prominent transcription factor that functions in a variety of physiologic processes including embryonic development, inflammation, immunity, and wound healing. Generally activated via growth factor and cytokine signaling, STAT3 can transcriptionally drive oncoproteins, pro-survival and pro-proliferative proteins as well as angiogenic factors, thereby contributing to cancer. As in most non-viral cancers, STAT3 is constitutively active in EBV-related B and epithelial cell cancers and in animal models of KSHV-cancers. Again, similar to non-viral cancers, STAT3 contributes to gammaherpesvirus (EBV and KSHV)-mediated cancers by driving cell proliferation, invasion and angiogenesis. Being herpesviruses, EBV and KSHV establish latency in humans with episodic lytic activation. Importantly, both viruses activate STAT3 almost immediately upon infection of primary cells. In the setting of infection of primary B cells by EBV, this rapidly activated STAT3 plays a key role in suppressing the DNA damage response (DDR) to EBV-oncogene triggered replication stress, thereby facilitating B cell proliferation and ultimately establishment of latency. STAT3 also contributes to maintenance of latency by curbing lytic activation of EBV and KSHV in latent cells that express high levels of STAT3. In this way, gammaherpesviruses exploit STAT3 to overcome cellular anti-proliferative and anti-lytic barriers to promote viral persistence. These investigations into gammaherpesviruses and STAT3 have simultaneously revealed a novel function for STAT3 in suppression of the DDR, a process fundamental to physiologic cell proliferation as well as development of cancer.

## Introduction

Epstein-Barr virus (EBV) and Kaposi's Sarcoma-Associated Herpesvirus (KSHV), members of the gammaherpesvirus subfamily, are oncogenic in humans. EBV (HHV4) infection is nearly ubiquitous; on the other hand, seroprevalence rates of KSHV (HHV8) range from less than 5% in northern and central Europe, North America and most of Asia to over 20% in the Middle East and the Mediterranean and 30–60% in Africa and the Amazon basin (Henke-Gendo and Schulz, 2004; Knipe and Howley, 2013). EBV and KSHV are both B lymphotropic and like other herpesviruses, establish latency with periodic lytic (re)activation to produce infectious virions (Knipe and Howley, 2013). This dual lifestyle, that includes latent and lytic phases, ensures persistence in a single host as well as in the human population.

While EBV persists as an asymptomatic infection in B cells of most individuals, its disease spectrum ranges from immunopathologic disorders such as infectious mononucleosis resulting from primary infection during early adulthood to endemic African Burkitt lymphoma (BL), Hodgkin lymphoma (HL), diffuse large B cell lymphoma (DLBCL), and nasopharyngeal cell carcinoma (NPC) in seemingly immunocompetent individuals and B cell lymphoproliferative disease (LPD) in immunocompromised hosts such as transplant and AIDS patients. Notably, EBV-related cancers are observed not only in B cells, the site of EBV latency, but also in epithelial cells, T cells and NK cells.

KSHV is linked most strongly to Kaposi's sarcoma (KS), a multifocal, angiogenic-inflammatory neoplasm that originates from vascular endothelial cells. Four varieties of KS have been described: Classic KS affecting middle aged men of Mediterranean and Eastern European descent, endemic African KS affecting children and young adults, post-transplant KS and AIDS-associated KS (Bhaduri-McIntosh, 2005). KSHV is also associated with 4 distinct lymphoproliferative disorders: primary effusion lymphoma (PEL; an AIDS-related body cavity B cell lymphoma), multicentric Castleman disease (MCD; primarily in HIV-infected individuals), MCD-associated plasmablastic lymphoma and HHV8-associated germinotropic lymphoproliferative disorder (Du et al., 2002; Cannon et al., 2003; De Paoli, 2004). Like EBV, KSHV-related neoplasms extend beyond B lymphocytes, specifically to endothelial cells.

## Features of gammaherpesvirus life cycle

EBV and KSHV genomes exist as episomes during latency with production of linear genomes packaged in capsid proteins as a result of lytic activation (Decker et al., 1996; Knipe and Howley, 2013). In healthy adults, between 1 and 50 per million circulating B cells are latently infected with EBV (Babcock et al., 1998). While herpesvirus genomes encode approximately 100 viral proteins, only a limited set is expressed during latency (Knipe and Howley, 2013). *In vitro* infection of primary B cells with EBV gives rise to indefinitely proliferating lymphoblastoid cell lines (LCL) that express all 9 latency proteins (i.e., type III latency). However, latently infected cells in healthy individuals express very few, if any, EBV proteins (type 0 latency) with increasingly more latency proteins expressed in BL and gastric carcinoma (type I), HL and NPC (type II) and LPD (type III) (Table 1). Importantly, limiting viral gene expression during latency provides a mechanism for escape from immune surveillance. Key latency proteins include LMP1, LMP2 and EBNAs for EBV, and v-FLIP, v-cyclin and LANA in the case of KSHV (Jenner et al., 2001; Knipe and Howley, 2013). These (and other) proteins serve multiple functions including promoting cell survival, proliferation, angiogenesis, invasion and immune modulation (Knipe and Howley, 2013).

**Table 1:** **Viral proteins expressed during different types of EBV latency**.

**EBV protein**	**EBNA1**	**EBNA2**	**EBNA3A**	**EBNA3B**	**EBNA3C**	**EBNA-LP**	**LMP1**	**LMP2A**	**LMP2B**
Latency type I	+								
Latency type II	+						+	+	+
Latency type III	+	+	+	+	+	+	+	+	+

Switch from latent to the lytic phase is an essential feature of the life cycle of herpesviruses. Lytic activation leads to regulated expression of a cascade of viral lytic genes of 3 kinetic classes: immediate early, early and late, accompanied by replication of viral genomes. Following packaging and release, virus particles can infect new cells in the same host and new hosts. While *in vivo* triggers for spontaneous lytic activation of gammaherpesviruses are a mystery, latently-infected EBV-positive (EBV^+^) B lymphoma cell lines and LCLs as well as KSHV-positive (KSHV^+^) PEL cell lines can be readily “induced” into the lytic phase in culture by phorbol esters, HDAC inhibitors, DNA methyltransferase inhibitors and immunoglobulin crosslinking (Knipe and Howley, 2013). Also, because EBV infects primary human B cells *in vitro* giving rise to LCLs, gammaherpesviruses, and in particular EBV, provide excellent models to study not only herpesvirus latency-to-lytic activation but also establishment of latency. Consequently, mechanisms underlying establishment of latency and activation of the lytic cycle have been studied in some depth in gammaherpesviruses and, in the last two decades, have revealed a prominent role for the cellular transcription factor STAT3 in latency, tumor-like properties of infected cells, and lytic activation of EBV and KSHV.

## STAT3

STAT3 is a well-studied member of the signal transducer and activator of transcription (STAT) family. Ligation of several cytokine (most notably IL6) receptors and growth factor receptors can activate STAT3; activation typically involves phosphorylation of a tyrosine residue (Y705) (Raz et al., 1994; Schindler and Darnell, 1995; Darnell, 1997, 2002). Phosphorylation can be mediated by receptor tyrosine kinases such as the Janus-activated kinase (JAK) family kinases or less frequently by non-receptor kinases such as Src (Schindler and Darnell, 1995; Silva, 2004). STAT3 can also be activated by phosphorylation of a serine residue (S727) (Hazan-Halevy et al., 2010). Activated STAT3 translocates to the nucleus and activates transcription of a large number of genes including *STAT3* itself; prominent among those are proproliferative and anti-apoptotic genes. STAT3 also plays critical roles in embryogenesis and immunity: deficiency of STAT3 results in death of mouse embryos by day 7 (Takeda et al., 1997) and in humans, STAT3 deficiency causes deficient T_H_17 cells, central memory T cells and memory B cells (Ma et al., 2008; Avery et al., 2010; Siegel et al., 2011). On the other hand, constitutive activation of STAT3, almost never associated with mutations in STAT3, is a feature of many human cancers (Yu and Jove, 2004).

In addition to activation in cancer cells, STAT3 can be activated by viruses such as HTLV1, Hepatitis B, Hepatitis C, varicella zoster virus, SV40, Friend virus and herpesvirus saimiri (Lee and Yun, 1998; Nakamura et al., 1999; Yoshida et al., 2002; Chung et al., 2004; Sarcar et al., 2004; Vultur et al., 2005; Ni et al., 2007; Sen et al., 2012). Many of these are oncogenic viruses, again underscoring the link between STAT3 and cancer. However, the link between STAT3 and oncogenic viruses has been investigated more comprehensively in the context of human gammaherpesviruses.

## STAT3 and gammaherpesvirus-related diseases

Activation state, typically phosphorylation at Y705, expression of STAT3 and presence of nuclear STAT3 have been examined in EBV-related cancers. Among B cell lymphomas, STAT3 was found to be constitutively active in post-transplant LPD and in spontaneous LCLs derived from patients with post-transplant LPD (Nepomuceno et al., 2002, 2003). STAT3 was also preferentially activated in EBV-positive DLBCL compared to EBV-negative DLBCL (Kato et al., 2014) and the presence of EBV in Hodgkin Reed-Sternberg cells correlated strongly with STAT3 expression (Garcia et al., 2003). Several studies have pointed out that STAT3 is also connected to epithelial cell cancers: cells from NPC have shown constitutively active and nuclear STAT3 (Chen et al., 2001; Hsiao et al., 2003; Buettner et al., 2006; Lui et al., 2009). Furthermore, EBV^+^ NPCs frequently had p-(Y705)STAT3, and phosphorylation at this position correlated with higher NPC stage (Liu et al., 2008).

The *in vivo* link between STAT3 and KSHV tumors is primarily derived from animal models. KSHV was found to increase STAT3 and NFκB levels in a tumor maintenance model in mice (Sun et al., 2015). Another study described the contribution of p-(Y705)STAT3 toward angiogenesis in KSHV-tumors in mice (Ma et al., 2013). Yet another study found that KSHV-encoded IL6 (vIL6) and mouse IL6 were both required for development of plasmacytosis and MCD-like pathology in a transgenic mouse model (Suthaus et al., 2012); IL6 is a potent activator of STAT3. Thus, there is substantial evidence for aberrant activation or increased levels of STAT3 in gammaherpesvirus-related cancers and cancer models. The following sections will describe investigations into STAT3 activation as well as the effects of STAT3 on host cellular functions and the life cycle of gammaherpesviruses.

## Mechanisms of STAT3 activation in the context of gammaherpesviruses

**Figure 1:**
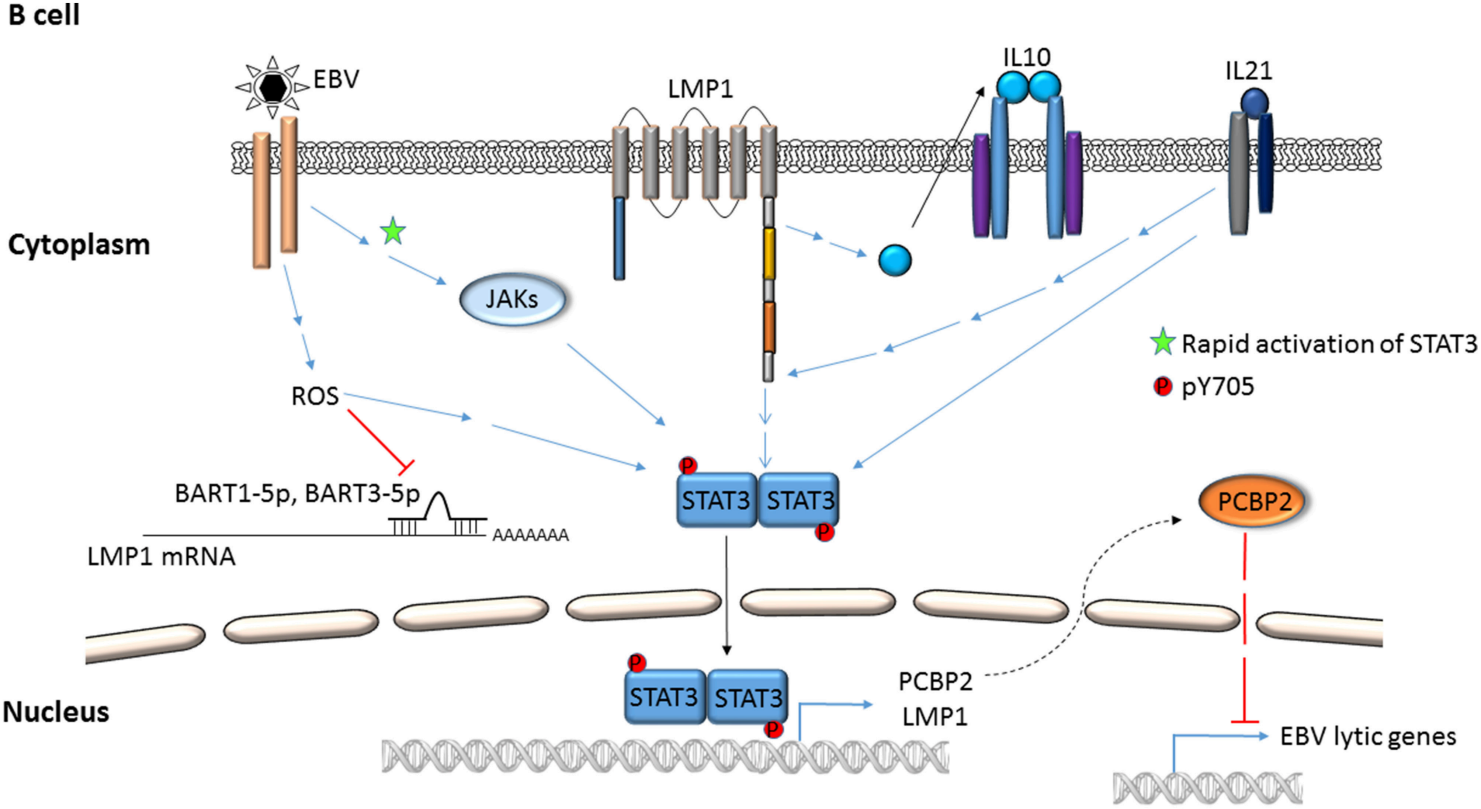
**Mechanisms by which EBV activates STAT3 in B lymphocytes**. EBV binding/entry rapidly activates STAT3 via Janus kinases. Additional modes of activation, i.e., phosphorylation of STAT3 at Y705 via ROS, cellular cytokines (IL-10, IL21), EBV LMP1 and EBV miRNAs are depicted. STAT3 can transcriptionally activate LMP1 and cellular PCBP2; PCBP2 represses EBV lytic genes.

**Figure 2:**
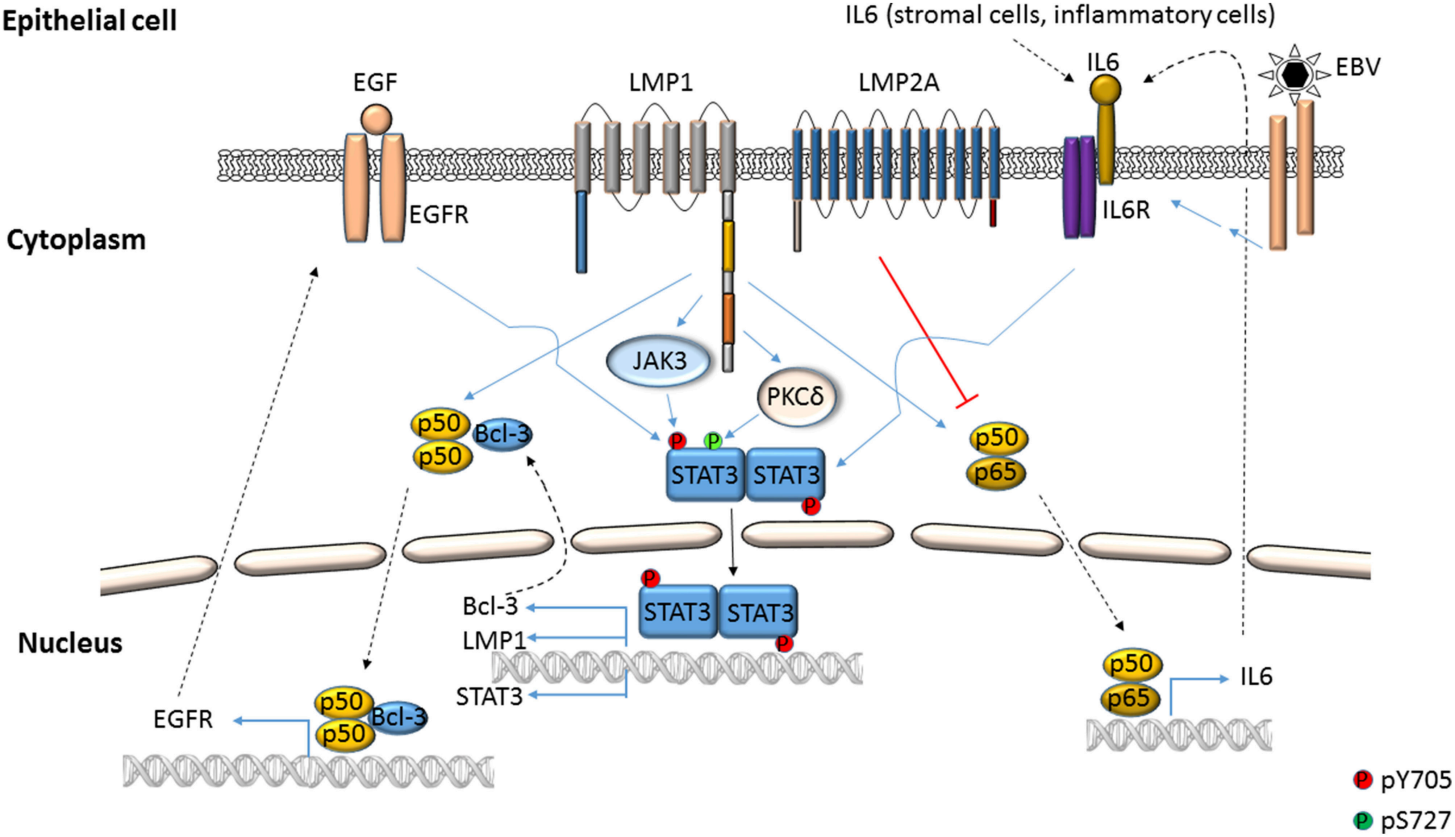
**EBV-mediated activation of STAT3 in epithelial cells**. STAT3 can be phosphorylated at Y705 by EBV LMP1 through the cooperation of cellular proteins such as NFκB, IL6, EGFR and Janus kinases. LMP1 can also cause phosphorylation of STAT3 at S727 through PKCδ. LMP2A can impair NFκB-mediated expression of IL6, thereby suppressing STAT3 activation.

**Figure 3:**
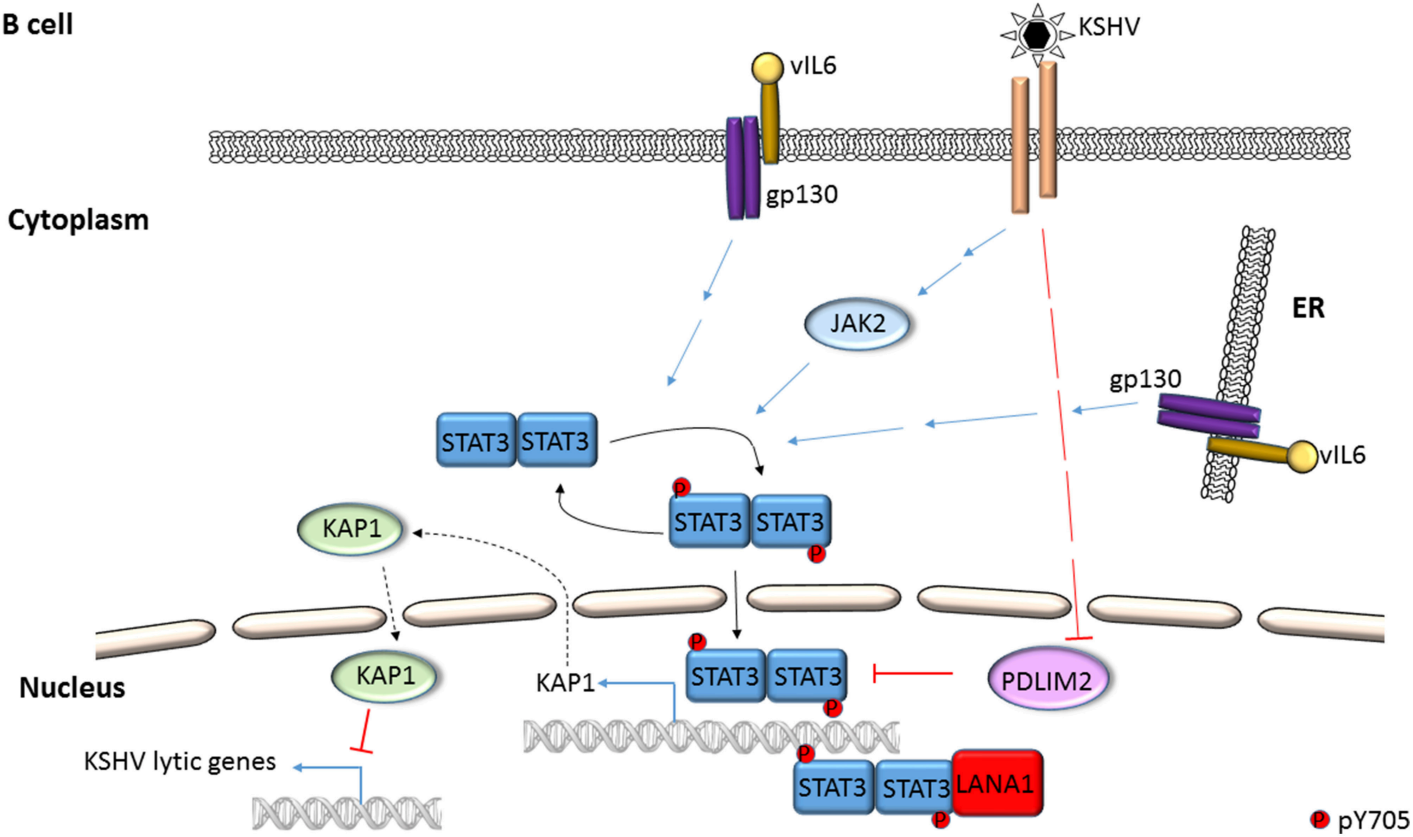
**Mechanisms of STAT3 activation in KSHV-infected B lymphocytes**. STAT3 is phosphorylated by Janus kinases and KSHV IL6 (vIL6) in PEL cells. Activated STAT3 can transcriptionally activate the *STAT3* gene while KSHV can repress cellular PDLIM2, an inhibitor of STAT3. KSHV LANA can serve as a transcriptional coactivator of STAT3. STAT3 represses KSHV lytic genes by transcriptional activation of the cellular corepressor KAP1.

**Figure 4:**
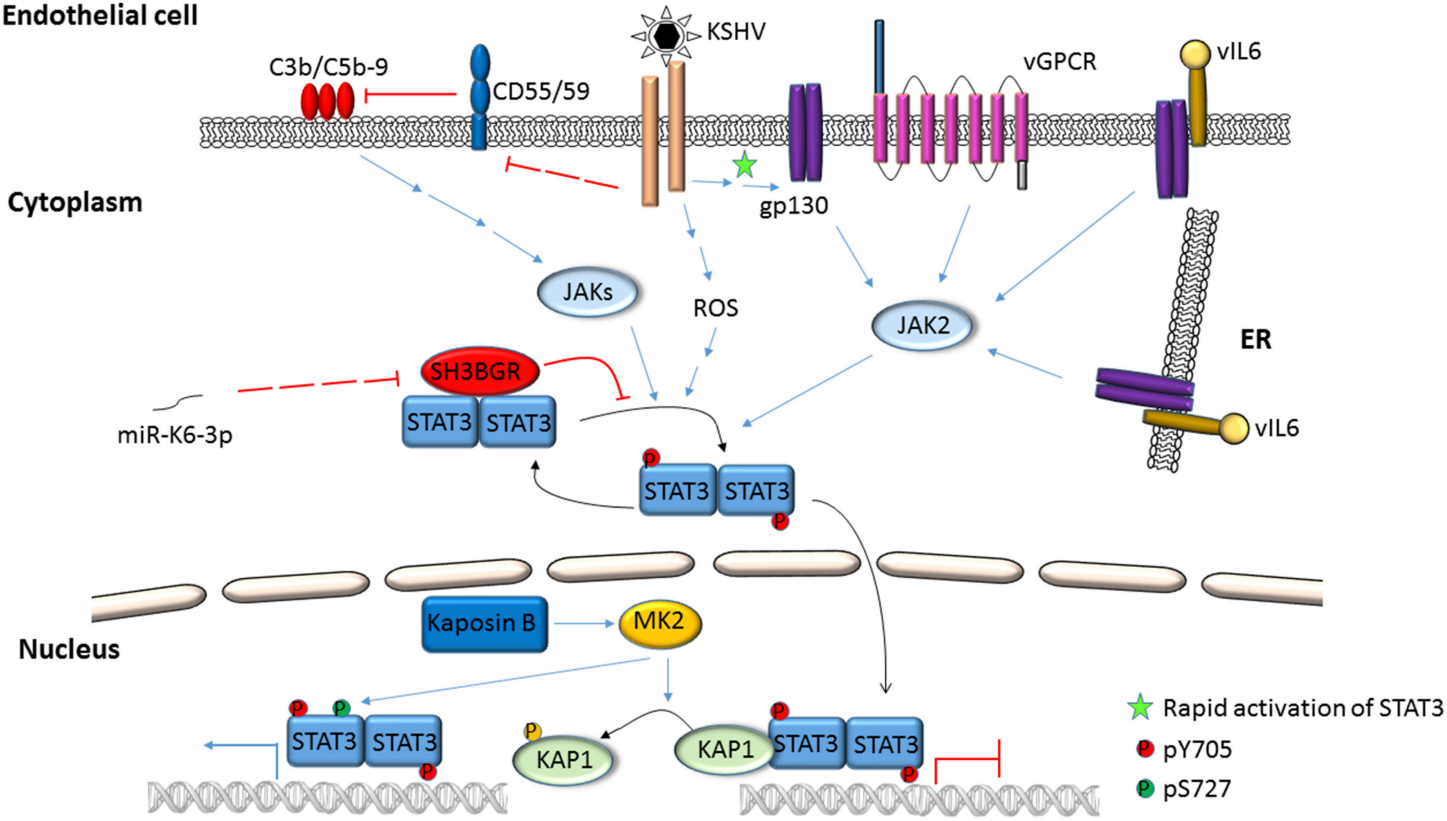
**Activation of STAT3 in KSHV-infected endothelial cells**. KSHV binding/entry into endothelial cells rapidly phosphorylates STAT3 at Y705 through Janus kinases. STAT3 can also be phosphorylated at S727 by the activities of viral Kaposin B through cellular MK2. Simultaneously, phosphorylation of cellular KAP1 by MK2 releases STAT3 from KAP1-mediated repression. Additionally, ROS, the alternate complement pathway and KSHV gene products such as vGPCR, vIL6 and miRNAs can also mediate phosphorylation of STAT3 at Y705.

Activation of STAT3 and its effects have been examined using virus infection models in culture, virus-infected/transformed cell lines (including those derived from patients with virus-related cancers), mouse models, and by selectively expressing viral proteins or their domains in uninfected cells. Mechanisms by which STAT3 is activated in the context of EBV and KSHV infection are depicted in Figures 1–**4**. Common themes between the two viruses include virus binding (or potentially virus entry) (Figures 1, **4**), reactive oxygen species (ROS) (Figures 1, **4**) and IL6 (Figures 2–**4**). Upon exposure of primary B cells to EBV or tert-immortalized microvascular endothelial (TIME) cells to KSHV, STAT3 was phosphorylated at Y705 within 30 min. This rapid phase activation by both viruses was dependent on virus binding and signaling via JAK but was independent of and preceded viral gene expression (Figures 1, **4**) (Punjabi et al., 2007; Koganti et al., 2014a). Another study showed that KSHV binding to DC SIGN activated STAT3 in dendritic cells (Santarelli et al., 2014). Notably, STAT3 activation in TIME cells was not mediated by IL6 (Punjabi et al., 2007).

ROS has been shown to activate STAT3 (Figures 1, 4) but much later after infection: 4 to 10 days after infection of primary B cells with EBV, ROS activated STAT3 while simultaneously inhibiting viral miRNAs that suppress LMP1 expression. Whether STAT3 and LMP1 activated each other in this setting was unclear (Chen et al., 2015). ROS was also shown to increase p-(Y705)STAT3 in a KSHV tumor model in mice (Ma et al., 2013). As for EBV-mediated activation of STAT3 via cellular IL6, this has been reported mainly in epithelial cells (Figure 2). NPC-associated fibroblasts, stromal cells and inflammatory cells produced IL6, likely resulting in increased IL6 in sera of NPC patients; some of these tumors also demonstrated increased expression of IL6 receptor, thereby enhancing STAT3 activation (Ma et al., 2008; Tsang et al., 2013; Zhang et al., 2013). IL6 expressed through the actions of LMP1 and NFκB has also been shown to activate STAT3 in NPC and HeLa cells (Chen et al., 2003; Tudor et al., 2012). In contrast to EBV, KSHV encodes a homolog of the cellular IL6 (i.e., vIL6) which has been found to activate STAT3 via the common gp130 subunit of IL6 receptor in endothelial cells and PEL cell lines; vIL6-gp130 signaling also originated in the endoplasmic reticulum (Morris et al., 2008, 2012; Cousins and Nicholas, 2013; Giffin et al., 2014, 2015; Wu et al., 2014) (Figures 3, 4).

Several virus-specific modes of STAT3 activation have also been observed. For example, IL21, known to activate STAT3, was found to cause LMP1 expression and STAT3 activation in EBV^+^ BL cells (Figure 1). Whether STAT3 was activated directly or via LMP1 remained unexplained (Kis et al., 2010). Recently, the alternate complement pathway was found to activate STAT3 in KSHV-infected endothelial cells and KS tumor cells (Figure 4). However, how these cells escaped death from complement activation was unclear (Lee et al., 2014).

Viral proteins have also been found to activate STAT3. Most remarkably, LMP1, through NFκB activation and IL6 expression activated STAT3 in NPC and HeLa cells (Figure 2). Activated STAT3 could in turn increase LMP1 expression via its TR promoter, driving a positive feed-forward loop in these cells. Based on these studies, the authors also proposed that in EBV^+^ cancers characterized by type II latency (i.e., lacking EBNA2), STAT3 may drive LMP1 expression (Chen et al., 2001, 2003). In an EBV-negative NPC line, LMP1 was found to activate STAT3 via JAK3 (Figure 2). Notably, LMP1, via PKCδ and ERK could also cause phosphorylation of STAT3 at S727. PKCδ also increased EGFR expression leading to increased p-(Y705)STAT3. This picture was further complicated by active STAT3-mediated increase in EGFR via Bcl-3, NFκB and PKCδ (Kung and Raab-Traub, 2008; Liu et al., 2008; Kung et al., 2011) (Figure 2). LMP2A, another EBV protein, was also shown to activate STAT3 in gastric cancer cells and fibroblasts (Hino et al., 2009; Nakaya et al., 2013). On the other hand, LMP2A could block STAT3 activation by inhibiting NFκB, thereby impairing LMP1 expression. This latter finding provided a mechanistic basis for the observation that NPC cells express LMP2A but very little LMP1 (Stewart et al., 2004).

Following infection of endothelial cells, KSHV Kaposin B was found to cause phosphorylation of KAP1 (a transcriptional corepressor that functions by recruitment of heterochromatin inducing factors) at S473 via MAP kinase-activated protein kinase 2 (MK2; a serine/threonine protein kinase). Phospho-KAP1 then derepressed *STAT3* transcription; MK2 simultaneously caused phosphorylation of STAT3 at S727 (King, 2013; Figure 4). In a separate study, KSHV was found to repress PDLIM2 by promoter methylation resulting in increased levels of STAT3 and NFκB (Figure 3). Importantly, suppression of PDLIM2, a putative tumor suppressor, is thought to contribute to tumor cell migration (Sun et al., 2015).

STAT3 function can also be modulated via protein-protein interaction. EBV EBNA2 and cellular EGFR have been shown to independently bind STAT3 and increase its transcriptional activity in epithelial cells (Lo et al., 2005; Muromoto et al., 2009). Interestingly, cellular SMRT (a transcriptional corepressor that facilitates recruitment of histone deacetylases to promoters) was shown to impair EBNA2-mediated STAT3 coactivation while EBNA2 interfered with SMRT and STAT3 interaction in EBV-negative epithelial cells (Ikeda et al., 2009). In the context of KSHV, LANA was shown to bind and cause transcriptional coactivation of STAT3 in PEL cells (Figure 3); similarly, ORF50 bound to and increased STAT3 transcriptional activity in epithelial cells and fibroblasts (Gwack et al., 2002; Muromoto et al., 2006). More recently, KSHV miR-K6-3p was shown to downregulate cellular SH3BGR (SH3 domain binding glutamate-rich protein), thereby relieving STAT3 from the inhibitory interaction with SH3BGR to cause enhanced activation of STAT3 (Figure 4; Li et al., 2016). Taken together, these studies underscore the complex relationships between STAT3, other cellular proteins and gammaherpesvirus proteins.

In summary, EBV- and KSHV-infection mediated activation of STAT3 can be divided into very early and late phases. Early activation is triggered by virus binding (or entry) and JAK-STAT signaling while late phase activation involves a multitude of cellular and viral proteins. Importantly, the functional consequences of STAT3 activation during the early phase appear to be distinct from activation at later times after infection. Late phase activation of STAT3 is important for virus persistence and potentiating cancer-like properties of infected cells. In comparison, the consequences of almost-instantaneous activation of STAT3 are not well-understood other than the critical role of EBV-activated STAT3 in rapidly crippling the DNA damage response (DDR) in B cells (described below).

## Contribution of STAT3 to cancer-related properties of gammaherpesviruses

Mice transgenic for keratin-promoter driven LMP1 and LMP2A developed squamous cell carcinoma with increased frequency; these lesions demonstrated high levels of STAT3 and ERK (Shair et al., 2012). In another study by the same investigators, LMP1-transgenic mice developed STAT3-dependent B-1a cell lymphomas after 12 months of age at a higher frequency compared to wild type mice (Shair et al., 2007). In NPC cell lines, STAT3 has been found to promote cell growth and invasion. These properties could be attributed to STAT3-mediated transcriptional activation of cellular genes such as MUC1, c-Myc, VEGF, and cyclin D1, all known for their tumor-promoting functions (Weber-Nordt et al., 1996; Kondo et al., 2007; Lui et al., 2009; Wang et al., 2010; Shair and Raab-Traub, 2012). STAT3 has also been shown to suppress cellular miR204 to increase cdc42 and NPC cell line invasion and metastasis (Ma et al., 2014). In a somewhat indirect mechanism in gastric cancer cells, LMP2A-activated STAT3 was found to increase DNMT1 levels which led to transcriptional repression of PTEN, a well-known tumor suppressor.

Activated STAT3 contributed to cell proliferation and angiogenesis in a KSHV tumor model in mice (Ma et al., 2013). Moreover, KSHV vIL6-transgenic mice developed human plasma cell-type MCD accompanied by STAT3 activation (Suthaus et al., 2012). In KSHV- infected culture systems, STAT3 contributed to endothelial cell survival, proliferation, migration, differentiation and angiogenesis as well as survival of PEL cell lines. These functions resulted from STAT3-mediated transcriptional activation of survivin, DNMT1 and CEACAM1 (a member of the carcinoembryonic gene family that participates in cell-cell adhesion). Furthermore, STAT3 was found to block autophagy in KSHV-infected dendritic cells (Aoki et al., 2003; Morris et al., 2008, 2012; Cousins and Nicholas, 2013; Santarelli et al., 2014; Wu et al., 2014; Giffin et al., 2015). Thus, STAT3 can mediate a multitude of pro-tumorigenic functions during gammaherpesvirus infection.

## A key role for STAT3 in establishment of EBV latency via blockade of DNA damage response signaling

Two sets of observations in the literature led to this line of investigation. First, most human cancers demonstrate constitutively active STAT3 (Yu and Jove, 2004); yet, whether STAT3 contributes during the early stages of cell transformation was not known. Second, contrary to expectations that sporadic cancers were triggered by driver mutations in genes belonging to the DDR category, majority of human sporadic cancers did not have such mutations; instead, most had driver mutations in cytokine and growth factor signaling pathways (Greenman et al., 2007). The realization that most of these signaling pathways activate STAT3 prompted the question: does STAT3 suppress the DDR to facilitate oncogene-driven cell proliferation?

**Figure 5:**
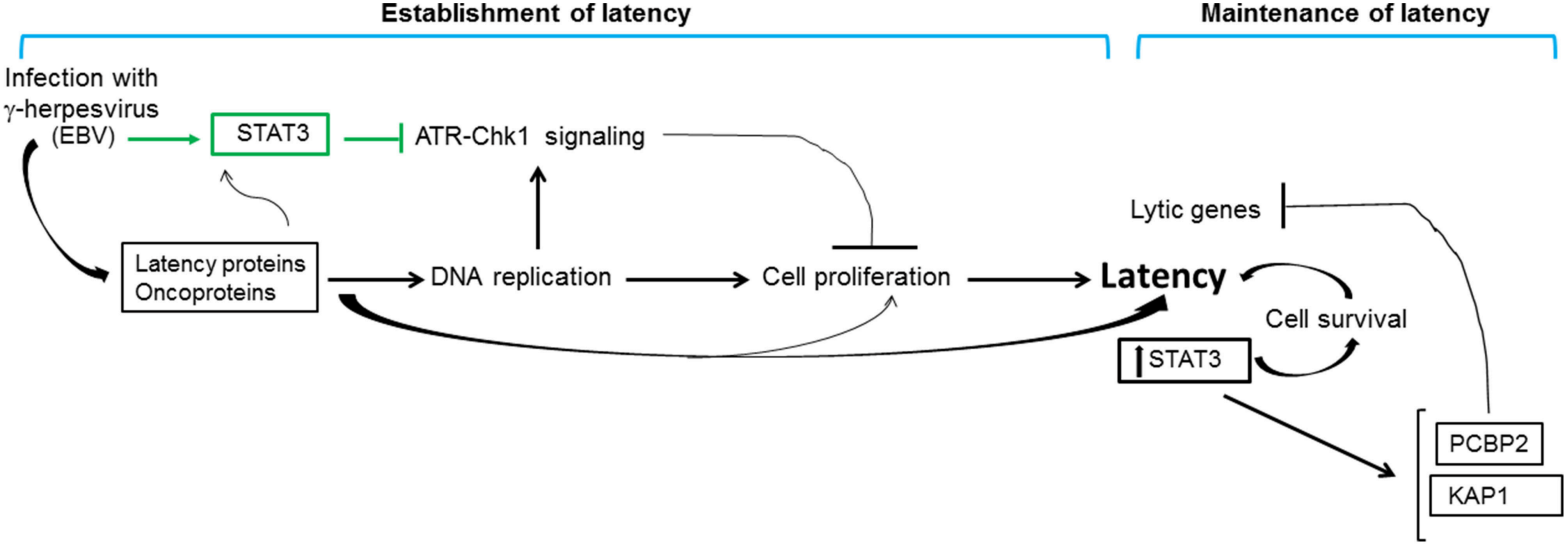
**Contribution of STAT3 to gammaherpesvirus persistence**. Infection of primary cells with gammaherpesviruses results in rapid activation of STAT3 (indicated in green). In the context of EBV infection, rapidly activated STAT3 blocks replication stress-induced cellular ATR-to-Chk1 signaling resulting in relaxation of the intra S phase cell cycle checkpoint; replication stress results from viral oncoprotein-driven cellular DNA replication. Relaxation of the intra S phase checkpoint ensures that infected cells do not undergo apoptosis or senescence in the early stages of infection, thereby promoting cell proliferation and establishment of viral latency. Latently-infected cells demonstrate high levels of STAT3 which contributes to cell survival and maintenance of latency. Furthermore, STAT3 transcriptionally activates cellular proteins PCBP2 and KAP1 that repress lytic genes, thus preventing loss of latency. The net effect of these STAT3-mediated activities is to promote gammaherpesvirus persistence.

Experiments to address the above question revealed that STAT3 was activated very rapidly (within 30 min) after exposure of primary B cells to EBV (Koganti et al., 2014a); a similar observation was also reported in KSHV-exposed endothelial cells (Punjabi et al., 2007). Activation of STAT3 resulted in activation of the effector caspase 7 but not caspase 3 or 6 in EBV-infected cells. Caspase 7 degraded the cellular protein claspin temporally before EBV oncogene-induced cellular DNA replication stress could be observed. Because claspin was absent, ATR-Chk1 signaling in response to replication stress was interrupted and the intra S phase cell cycle checkpoint was relaxed. Intra S phase checkpoint relaxation safeguarded against senescence or apoptosis despite replication stress. Continued cell proliferation ensured outgrowth of latently infected EBV^+^ cell lines (Koganti et al., 2014a,b). Therefore, during the very early stages of infection, EBV uses a cellular mechanism (involving STAT3, caspase 7 and claspin) to block ATR-Chk1 signaling, thereby ensuring that cell proliferation would endure while viral latency gene expression gained traction (Figure 5). Key components of this mechanism were confirmed in circulating naturally-infected proliferating B cells from patients with primary EBV infection. This set of experiments exemplifies the use of viruses to uncover novel cellular mechanisms. In doing so, a new function for STAT3 in mitigation of replication stress-induced DDR signaling was also discovered—a function that may contribute to cell cycle checkpoint recovery during physiologic cell proliferation as well as implicating STAT3 as a molecular switch used by cancers to turn down DDR signaling during oncogene-induced replication stress.

## STAT3 as a cellular rheostat between gammaherpesvirus latent and lytic cycles

A long-standing observation in the herpesvirus field has been that only a fraction of latently infected cells responds to lytic cycle inducing triggers. Indeed, only about 50% of B cells latently infected with EBV respond to lytic inducing agents at any time (Bhaduri-McIntosh and Miller, 2006). While this property of partial permissiveness ensures herpesvirus persistence, it simultaneously hinders the effectiveness of oncolytic therapeutic approaches that pharmacologically activate the viral lytic cycle and kill lytic cells using anti-viral agents. Sorting single EBV^+^ lytic cells from cells refractory to lytic triggers resulted in the identification of STAT3 as a key cellular switch between latent and lytic phases. Specifically, a high level of STAT3 hindered the transition of EBV and KSHV to the lytic phase despite exposure to lytic cycle inducing triggers (Daigle et al., 2010; Hill et al., 2013; King et al., 2015; Koganti et al., 2015). In EBV-infected cells, STAT3-mediated resistance to lytic cycle activation was executed via PCBP2, a cellular multifunctional poly(rC)-binding protein (Figures 1, 5; Koganti et al., 2015). In KSHV-infected cells, STAT3 inhibited viral lytic gene transcription via KAP1, a well-known transcriptional co-repressor (Figures 3, 5; King et al., 2015). Importantly, STAT3-mediated regulation of lytic susceptibility may be broadly utilized by human herpesviruses as STAT3 was also shown to restrict lytic activation of HSV1 (Du et al., 2013).

The presence of high levels of STAT3 in latent/refractory cells serves two functions. From a virologic viewpoint, limiting lytic activation to a fraction of infected cells ensures herpesvirus persistence in the refractory population. On the other hand, restricting lytic activation may be viewed as a cellular anti-viral strategy, since the lytic phase is responsible for significant pathology particularly for alpha and betaherpesviruses.

## Conclusion

Two decades of investigations have revealed a complex relationship between the cellular proto-oncogene STAT3 and the highly prevalent human gammaherpesviruses. As with many physiologic and pathologic conditions, STAT3 can be activated by signaling through cytokine- and growth factor-receptors. STAT3 can also be activated by herpesvirus binding, viral proteins and ROS. Further, STAT3 can be transcriptionally regulated. Activation and increased expression of STAT3 contribute to growth promoting, invasive and angiogenic properties of tumor cells in tissue culture and animal models. Apart from its well-described tumorigenic properties, STAT3 orchestrates herpesvirus persistence. By interfering with DDR signaling immediately after EBV infection of B cells, it ensures establishment of latency. By restricting lytic cycle activation in latently infected EBV^+^, KSHV^+^ and HSV^+^ cells, STAT3 also ensures maintenance of latency. Thus, the net effect is to promote herpesvirus persistence in the host (Figure 5). Continued investigation into how STAT3 mediates these novel functions in virus-infected cells is likely to uncover new mechanistic links between cellular and viral machineries that may be exploited for therapeutic applications.

## Author contributions

All authors listed, have made substantial, direct and intellectual contribution to the work, and approved it for publication.

### Conflict of interest statement

The authors declare that the research was conducted in the absence of any commercial or financial relationships that could be construed as a potential conflict of interest.
